# Laparoscopic Hiatal Hernia Repair: Short-Term Results From Yemen in a Resource-Limited Setting

**DOI:** 10.7759/cureus.78010

**Published:** 2025-01-26

**Authors:** Burkan Nasr, Abdulhakim Altamimi, Badr Altohari, Ali Obad, Ghassan Ali, Ahmed Alaidaroos, Salem Barabaa, Sumiah Bahanan, Gubran Othman

**Affiliations:** 1 Department of Surgery, University of Aden, Aden, YEM; 2 Department of Surgery, Aden German International Hospital, Aden, YEM

**Keywords:** complications, gerd, hiatal hernia repair, laparoscopic surgery, nissen fundoplication

## Abstract

Background

The laparoscopic minimally invasive surgery with anti-reflux procedure is the preferred method for hiatal hernia repair, showcasing a noticeable decrease in surgery-related morbidity and mortality. This study aimed to investigate various elements and variables that could affect and enhance the advantages of minimally invasive surgery for hiatal hernias and minimize the chances of complications occurring both during and after laparoscopic repair with fundoplication for hiatal hernia.

Methods

Hiatal hernia repair with fundoplication as anti-reflux surgery was conducted to evaluate perioperative and postoperative outcomes at Aden Hospital between 2023 and 2024. The inclusion criteria included patients with hiatal hernia and a positive history of gastroesophageal reflux treated with laparoscopic minimally invasive hernial repair involving anti-reflux procedures such as laparoscopic Nissen fundoplication or Dor fundoplication. Data on baseline population characteristics, including age and gender, as well as hernia types (type 1, 2, 3, and 4), hernia size, and the surgical techniques used were collected. Information regarding operative duration, intraoperative complications, postoperative complications, and length of hospital stay was also gathered. Follow-up assessments were conducted at one, three, six, and 12 months.

Results

From 2023 to 2024, a total of 21 individuals underwent minimally invasive laparoscopic hiatal hernial repair, which included 12 (57%) females and nine (43%) males, with an average age of 55 years (ages ranging from 35 to 80 years). Symptoms of gastroesophageal reflux such as heartburn manifested in 18 (85%) patients. Three (14%) patients had abdominal surgery history. The types of hiatal hernia observed were as follows: 12 patients had type 1, five had type 2, three had type 3, and one had type 4. Conversion from laparoscopic surgery to open surgery was performed in one case (4.7%). Sixteen (76.1%) patients had laparoscopic hiatal hernia repair combined with Nissen fundoplication, three (14.2%) patients had Heller myotomy with Dor fundoplication, and two (9.5%) patients underwent sleeve gastrectomy along with hiatal hernia repair. The average duration of the operation was 116 ± 60 minutes, while the average length of hospital stay was 3 ± 1.5 days. There was one (4.7%) patient with intraoperative complication (pneumothorax), and 15 (71.4%) patients were free of postoperative complications; however, four (19%) patients complained of postoperative flatulence and abdominal distension, one (4.7%) patient complained of transient recurrent reflux and dysphagia, and one (4.7%) patient had aspiration pneumonia and death. Recurrent hiatal hernia was not detected during follow-up at three to 12 months after laparoscopic surgery.

Conclusions

Laparoscopic hiatal hernia repair with anti-reflux surgery can be successful in resource-limited settings, providing an effective and safe option for managing hiatal hernias and alleviating gastroesophageal reflux disease.

## Introduction

A hiatal hernia occurs when the gastroesophageal junction, the upper part of the stomach, or other abdominal viscera pushes through weakened muscle tissue of the diaphragm into the chest cavity. This condition is frequently linked to symptomatic gastroesophageal reflux disease (GERD). When compared to the open surgical technique, the laparoscopic approach for repairing hiatal hernias has gained acceptance as a surgical treatment and is associated with fewer surgery-related complications and fewer hospitalization duration days [1]. The laparoscopic fundoplication is the established surgical procedure for addressing hiatal hernia with GERD symptoms, and it should be preferred over the open alternative. This is due to its ability to provide enhanced visualization of the hiatal region, shorter hospital stays, and, while maintaining similar effectiveness, superior cosmetic outcomes with lower morbidity and mortality rates (0.04% vs. 0.2%) [2,3].

Accurately determining the prevalence of hiatal hernia is challenging, as asymptomatic cases often go unnoticed. Nevertheless, symptomatic hernias should be evaluated in connection with GERD concerning its pathophysiology, given the increasing global incidence of GERD based on diagnostic rates [4,5].

Medical management, such as proton pump inhibitors, is typically the first-line approach to symptom control; however, surgical interventions may be warranted depending on symptom severity and hernia type. Laparoscopic repair has gained popularity recently due to its numerous advantages [6,7].

Various factors contribute to the inadequacy of primary closure for hiatal hernia. Tension overload leads to repair failure, especially in cases where the hernia defect with an intrathoracic stomach is substantial. Also, the diaphragmatic hiatal between the crus is usually made of weak muscles that are exposed to a lot of strain from actions like breathing and coughing [8,9].

Hiatal hernia and GERD are recognized complications of obesity. Recent research suggests that a proactive approach to laparoscopic hiatal hernia repair in obese patients undergoing bariatric surgery may result in reduced GERD symptoms and lower rates of hiatal hernia recurrence [10,11].

Performing laparoscopic hiatal hernia repair is a complex procedure that demands proficiency in advanced minimally invasive surgical techniques, as well as a deep understanding of esophageal and stomach anatomy and physiology. When executed correctly, the operation should lead to a high rate of symptom resolution with minimal complications, even in elderly patients with multiple medical conditions.

In this article, we explain our experiences with advanced laparoscopic surgery for the management of hiatal hernias and review the results of our operations.

## Materials and methods

This study involved 21 patients diagnosed with a hiatal hernia who underwent laparoscopic surgery between 2023 and 2024. All patients provided informed consent for participation. Data pertaining to baseline demographic characteristics of the population, such as age, gender, type of hernia (type 1, 2, 3, and 4), size of hernias, and the surgical methodologies employed, were compiled. Additionally, information on the duration of the operation, intraoperative complications, postoperative complications, and length of hospitalization was gathered.

Standard preoperative assessments, including physical examinations and laboratory tests, were conducted; moreover, esophagogastroduodenoscopy and chest and abdominal computed tomography (CT) scans were carried out before the procedure. However, esophageal manometry and barium esophagography were not included or mandated in our study. Surgical intervention was indicated due to symptoms like reflux or obstruction. Patients were required to provide consent for the surgery after receiving a comprehensive explanation of the untreated hiatal hernia's natural progression and the pertinent details of the surgical procedure, as well as the associated risks.

Hiatal hernias were categorized into four types based on their distinctive features [12]. Hiatal hernia type 1 involves sliding the gastroesophageal junction into the thoracic cavity. In hiatal hernia type 2, referred to as para esophageal or rolling hiatal hernias, the gastroesophageal junction is in a normal position in the abdomen cavity while the upper part of the stomach is herniated up through the hiatus adjacent to the esophagus. Hiatal hernia type 3 commonly features the herniation of both the gastroesophageal junction and the stomach through esophageal hiatus. Lastly, hiatal hernia type 4 is characterized by the thoracic displacement of the stomach and nearby organs. An upper gastrointestinal series using barium was not routinely conducted postoperatively; instead, early initiation of oral feeding was based on the patient's symptoms and clinical stability. Following the surgery, all patients were examined at the outpatient clinic one to two weeks later for a comprehensive evaluation of their condition and symptoms. Subsequent follow-up appointments were scheduled at one month, three months, six months, and 12 months post surgery.

This study examined the medical traits, surgical factors, and postoperative results and consequences for every patient.

Surgical techniques

Patient Positioning

The patient's head was elevated to approximately 45 degrees. This position enhances visualization of the upper abdomen and facilitates retraction of the liver.

Trocar Placement

Five trocars were placed: two 10-12 mm trocars, including a supraumbilical larger trocar that likely accommodates the primary surgical instruments, and a left pararectal placed along the left side of the rectus abdominis muscle for inserting the camera. Two 5 mm trocars, including right and left subcostal, which are smaller trocars typically used for auxiliary instruments such as graspers or suction/irrigation devices. One 5 mm trocar (epigastric) specifically for introducing a "liver snake retractor." The insufflated pressure was 12-15 mm Hg, with a rate of 5-6 liter/minute.

**Figure 1:**
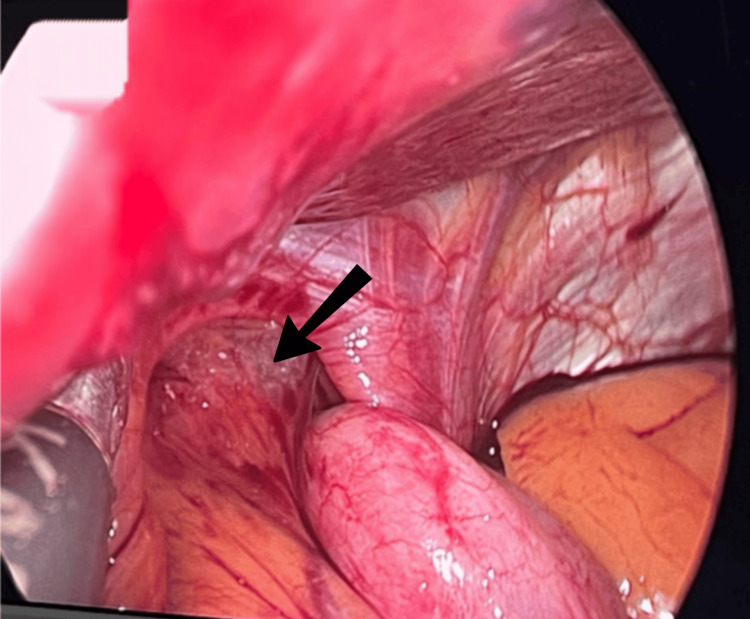
Big size (arrow) hiatal hernia (type 3), with gastroesophageal junction and gastric fundus above the diaphragm.

All the procedures were conducted by the same surgeon. Complete dissection and reduction of the hernia sac, along with mobilization of the distal esophagus, were undertaken. A tension-free crural repair was performed, followed by the creation of an effective anti-reflux fundoplication. The initial phase of the procedure involved reducing the hernia content within the abdomen, and subsequently, a bipolar energy instrument (LigaSure™, Medtronic, Dublin, Ireland; Harmonic, Ethicon, Raritan, NJ) was used to divide the short gastric vessels near the fundus (Figure 1).

**Figure 2:**
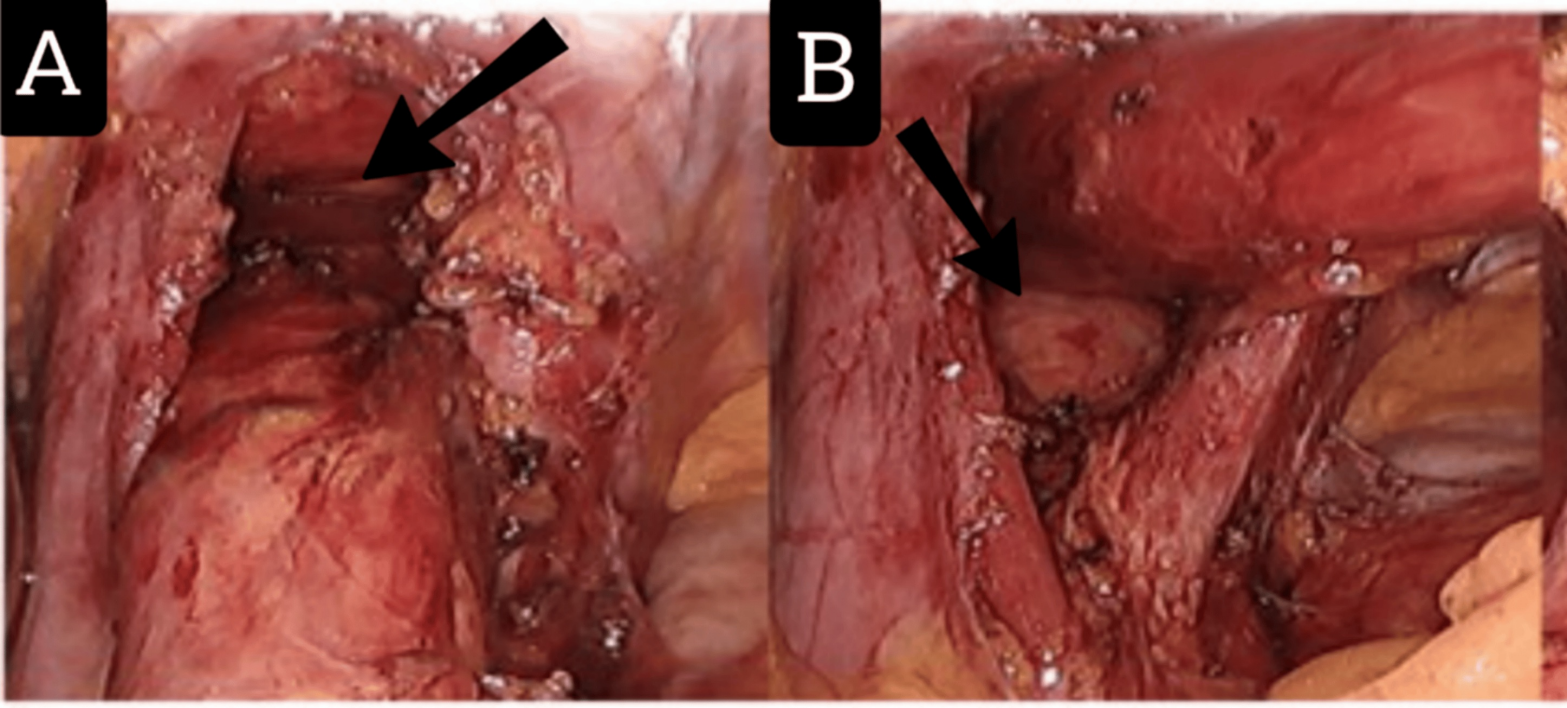
Sac removal of the hiatal hernia with dissection of the hiatal opening and crural. (A) Dissection from above (arrow). (B) Dissection from below (arrow).

**Figure 3:**
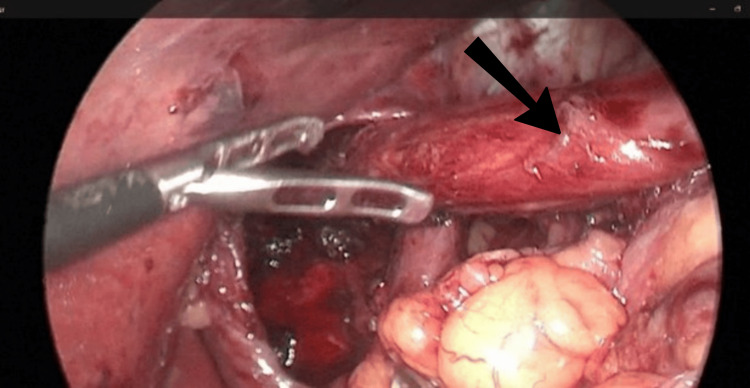
Adequate esophageal length below the diaphragm after mediastinum dissection (arrow).

**Figure 4:**
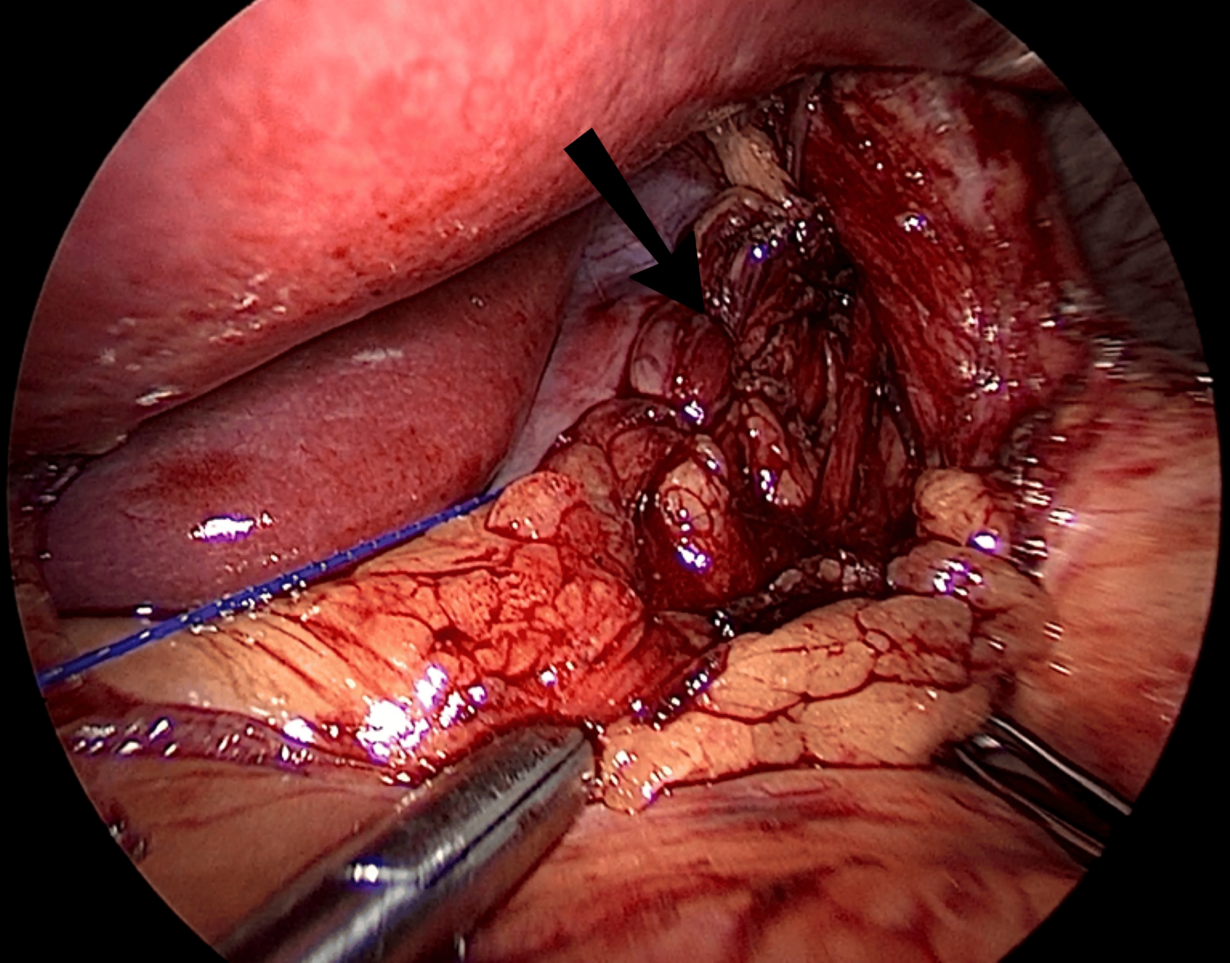
Posterior crural re-approximation (arrow).

Subsequently, the hernial sac was excised from all esophageal attachments (as depicted in Figure 2). This was done to increase the intra-abdominal length of the esophagus and reposition the gastroesophageal junction to its normal anatomical position. This was achieved by careful dissection around the esophagus within the mediastinum (Figure 3). Retroesophageal crural primary closure was then carried out by approximating the crura using nonabsorbable barbed sutures (2/0 or 3/0 V-Loc™, Medtronic or Stratafix™, Ethicon) as continuous stitches or three to five interrupted nonabsorbable sutures such as Prolene or Ethibond (Ethicon) (Figure 4).

In some instances, after hiatal hernia repair, a 360° fundus of the stomach encircles the esophagus, securing it with three interrupted Ethibond stitches. Nonetheless, the wrap was sutured to the esophagus using proximal stitches, this is called Nissen fundoplication. It is generally preferred for more severe and persistent GERD, as it provides a more complete wrap around the esophagus, offering stronger reflux control.

**Figure 5:**
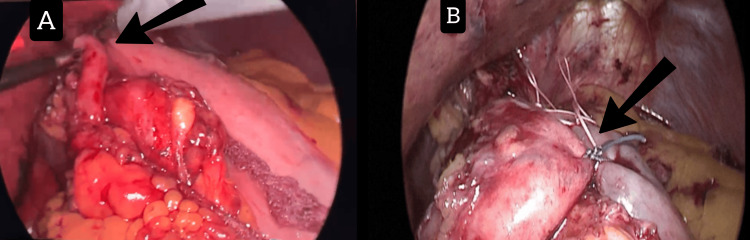
(A) Tension-free fundoplication (arrow). (B) Complete Nissen fundoplication (arrow).

For certain cases, Dor fundoplication entailed wrapping the stomach fundus 180° around the esophagus post hiatoplasty and affixing the wrap to the left and right crus (Figure 5 and Video 1).

**Video 1:** Hiatal hernia repair (HHR) surgery procedure by Dr. Burkan Nasr.

In cases of sleeve gastrectomy, the primary closure of the hiatal hernia was successfully achieved by approximating the crura with nonabsorbable barbed sutures (2/0 or 3/0 V-Loc™, Medtronic or Stratafix™, Ethicon).

Statistical analysis

Data were reported as percentage, median, mean, or range, as stated in the article. Statistical evaluations for this study were conducted using IBM SPSS Statistics version 19 for Windows (IBM Corp., Armonk, NY).

## Results

Laparoscopic hiatal hernia surgery was done for 21 patients with an average age of 55 years, including nine (43%) males and 12 (57%) females. The average body mass index was 35.23. Preoperative gastroesophageal reflux and other hiatal hernia symptoms were present in 18 (86%) patients. In five patients, hiatal hernias were discovered during laparoscopic surgery for achalasia and bariatric surgery (three and two, respectively). Three patients had a history of previous abdominal surgery: two patients underwent laparoscopy and one underwent laparotomy. Type 1 hiatal hernia was the most common (57%), followed by type 2 hiatal hernia (23%). Sixteen (76%) patients underwent hiatal hernia repair with Nissen fundoplication, three (14%) patients underwent hiatal hernia repair with Dor fundoplication after Heller myotomy for esophageal achalasia, and two (9%) patients underwent hiatal hernia repair with sleeve gastrectomy. The mean operative time was 116.90 (range = 150-100) minutes, the average size of the hiatal hernia was about 6 cm, and the mean length of hospital stay was three (range = 5-3) days. One case with severe adhesion, large liver, and big hernial sac was converted from laparoscopic to open upper midline laparotomy (Tables 1, 2).

**Table 1:** Demographic and baseline preoperative data.

Variables	Total no. of patients: 21
Age per year (mean) (range)	55 (35-80)
Gender, male: female (%)	9:12 (43%-57%)
Body mass index (kg/cm^2^) (mean)	35.23
Preoperative symptoms	
Heartburn or epigastric pain (%)	18 (86%)
Dysphagia or dyspepsia (%)	4 (19%)
No symptoms (%)	3 (14%)
Proton pump inhibitors (%)	18 (86%)
Prior abdominal surgery (%)	3 (14%)

**Table 2:** Operative data.

Variables	Total no. of patients: 21
Type 1 hiatal hernia	12 (57%)
Type 2 hiatal hernia	5 (23%)
Type 3 hiatal hernia	3 (14%)
Type 4 hiatal hernia	1 (4%)
Size of hiatal hernia (cm) (mean, range)	6 (10-2)
Hiatal hernia repair with Nissen fundoplication	16 (76%)
Hiatal hernia repair with Dor fundoplication	3 (14%)
Hiatal hernia repair with sleeve gastrectomy	2 (9%)
Operative time in minutes (mean, range)	116.90 (150-100)
Conversion to laparotomy	1 (4%)
Postoperative complications	6 (28%)
Length of hospital stay in days (mean, range)	3 (5-3)

Complications and follow-up summary are illustrated in Table 3. The median postoperative follow-up was six months (range = 3-12 months). There was one (4%) patient with intraoperative complication (pneumothorax), and 15 (71.4%) patients were free of symptoms postoperation; however, six (28%) patients had postoperative complications at outpatient follow-up: four (19%) patients had flatulence and abdominal distension and one (4%) patient complained of transient recurrent reflux and mild dysphagia symptoms; these symptoms were well-controlled with medication and remained free of abnormal imaging findings. One (4%) patient died due to postoperative aspiration pneumonia. Recurrent hiatal hernia was not detected during follow-up at three, six, and 12 months after laparoscopic surgery.

**Table 3:** Complications and follow-up summary. Data are presented as percentage (%) of patients with abdominal distension, heartburn, dysphagia, wound infection, esophageal injury, pneumonia, pneumothorax, recurrence, and death. Median and range are used for the duration of follow-up (months).

Variables	Total no. of patients: 21
Abdominal distension, bloating	4 (19%)
Heartburn or epigastric pain	1 (4%)
Dysphagia	1 (4%)
Wound infection	0 (0/21)
Esophageal injury or leaks	0 (0/21)
Pneumonia	1 (4%)
Pneumothorax	1 (4%)
Recurrence	0 (0/21)
Death	1 (4%)
Duration of follow-up (months) (median, range)	6 (12-3)

## Discussion

In laparoscopic hiatal hernia repair in resource-limited settings, the surgeon's experience and adapting surgical techniques to optimize outcomes with limited resources are crucial, utilizing basic laparoscopic instruments and minimizing the need for specialized or expensive equipment, concentrating on the core elements of the repair, including reduction of the hernia sac and cruroplasty (repairing the diaphragmatic opening) with or without fundoplication.

The presentation of a hiatal hernia can exhibit significant variation. As reported by Mehta et al., a hiatal hernia may remain asymptomatic or it may manifest with symptoms of reflux or obstruction [13]. Although diagnosing asymptomatic hiatal hernia in patients poses challenges, there has been a recent rise in the diagnosis rate, corresponding to the increased frequency of routine health screenings [14]. According to Grant et al., GERD is a major clinical manifestation of hiatal hernia. Pharmacological intervention has become the primary approach for symptom relief due to the inherent complications associated with open surgical procedures. Nonetheless, the adoption of laparoscopic surgical techniques has greatly reduced the complications associated with surgical procedures. Furthermore, it is more effective than pharmacotherapy in providing lasting symptom relief and offering economic benefits [15].

In relation to hiatal hernia, some advocate for a cautious observation as a viable initial approach for managing patients with asymptomatic or mildly symptomatic paraesophageal hernias (PEHs). Nonetheless, the majority of specialists contend that genuinely asymptomatic PEHs are uncommon. Furthermore, the annual probability of conversion from asymptomatic hiatal hernia to symptomatic was approximately 14%, as documented by Stylopoulos et al. [16]. These factors were meticulously explained to all patients in our study before obtaining their informed consent for surgical intervention.

Anand et al. highlight that obesity increases the risk of hernial recurrence and that bariatric surgery can address obesity, potentially reducing this risk [17]. Kohn et al. emphasize that according to the current guidelines, all hiatal hernias identified during bariatric surgeries should be repaired [1]. It is noteworthy that during bariatric procedures, two patients in our care were incidentally found to have small sliding hiatal hernias (≤2 cm in diameter), which were repaired simultaneously with the bariatric surgery.

The laparoscopic correction of hiatal hernias has emerged as the preferred surgical method due to its reduction in discomfort, complications, and hospitalization duration compared to the traditional open technique [18]. Recent advancements in the laparoscopic approach and accumulated expertise may now potentially decrease the recurrence rate while upholding the aforementioned benefits [19,20]. Karmali et al. documented a recurrence rate of about 9% with both open and laparoscopic procedures [21]. Notably, no patients in this study exhibited a recurrent anatomical hiatal hernia, as there was a substantial decrease in the severity of most symptoms postoperatively. However, given that postoperative upper gastrointestinal assessments were not done in this study, there is a possibility that some patients with recurrent hiatal hernias could be asymptomatic. This is consistent with the study conducted by Oelschlager et al. [22]. The presence or absence of symptoms does not reliably indicate the recurrence of a hiatal hernia.

Pneumothorax can occur as a complication during laparoscopic hiatal dissection. A study by Phillips et al. documented 22% (11/50) of patients with pneumothorax. One patient in this study experienced pneumothorax coupled with transient hypotension, necessitating the placement of a small-bore chest tube on the right side for 48 hours [23].

Dallemagne et al. reported that abdominal distension and flatulence (wind problems) are common side effects after hiatal hernia surgery with fundoplication. In our study, four (19%) patients experienced increased flatulence and abdominal distention postoperatively. These symptoms resolved with symptomatic treatment within the first three months of follow-up [2].

Hunter et al. demonstrated that postoperative dysphagia predominantly stems from issues related to hiatal closure [24]. Additionally, a notable discovery from our investigation is the absence of prolonged dysphagia. While one (4%) patient experienced initial dysphagia with mild reflux symptoms following laparoscopic hiatal hernia repair, it eventually subsided in the first month with long-term use of proton pump inhibitors. Some studies suggest that routine use of bougies (dilators) during hiatal hernia surgery may not be necessary to prevent dysphagia. This means that using a bougie might not significantly reduce the risk of dysphagia after surgery. If a bougie was not used, surgeons likely relied on other techniques to ensure proper closure of the hiatal opening. These might include careful visual inspection of the area during the operation to ensure adequate closure and prevent constriction of the esophagus; also, with experience and judgment, surgeons develop a sense of appropriate tension based on their training and surgical experience [24].

Dallemagne et al. and Morino et al. demonstrate low mortality (0.2%) post laparoscopic hiatal hernia repair [2,3]. In our study, one patient died postoperatively. This 80-year-old patient had multiple comorbidities, including diabetes, stroke (cerebrovascular accident), and dementia. He presented with a large hiatal hernia and a history of recurrent aspiration pneumonia. On the fifth postoperative day, he developed massive aspiration pneumonia and subsequently died.

Undoubtedly, surgeons performing laparoscopic surgery for paraesophageal hernias require significant training and experience to maintain an acceptable recurrence rate, with a minimum of 20 cases performed each year [25]. Patients with severe or persistent reflux may benefit more from the stronger seal provided by Nissen fundoplication as Toupet fundoplication may be less effective in controlling severe acid reflux and postoperative dysphagia. Surgeons should choose between the two surgical techniques depending on their experience and patient factors, such as esophageal motility disorders.

The limitations or restrictions of this study included its restricted size and the brief duration of the investigation period. Nevertheless, the frequency and diagnostic rates of hiatal hernia are still inadequately documented in our nation. Thus, there exists a significant lack of literature addressing the surgical treatment of this condition; hence, we endeavored to share our experiences in laparoscopic hiatal hernia surgery.

## Conclusions

Laparoscopic hiatal hernia surgery is an anatomically challenging procedure that requires a significant learning curve. However, with access to a good laparoscopic setup and an experienced surgeon, it becomes a feasible technique. This method generally results in satisfactory outcomes and fewer surgical complications, with improved gastroesophageal reflux symptoms. Laparoscopic hiatal hernia repair in resource-limited settings can be successful by adapting techniques to minimize the need for specialized equipment, with continued equipment maintenance and training and upskilling of existing staff to assist with laparoscopic procedures to optimize surgical workflow, ensuring adequate postoperative care for potential comorbid patients.
